# Cytoguardin: A Tryptophan Metabolite against Cancer Growth and Metastasis

**DOI:** 10.3390/ijms22094490

**Published:** 2021-04-26

**Authors:** Kenneth K. Wu

**Affiliations:** 1 Institute of Cellular and System Medicine, National Health Research Institutes, Zhunan Town 35053, Taiwan; kkgo@nhri.org.tw; Tel.: +886-37-246-166 (ext. 37501); Fax: +886-37-587-408; 2 Institute of Biotechnology, College of Life Science, National Tsing-Hua University, Hsinchu 30013, Taiwan

**Keywords:** cytoguardin, 5-methoxytryptophan, cancer growth, cancer metastasis, cyclooxygenase-2, matrix metalloproteinase, epithelial mesenchymal transition

## Abstract

Cytoguardin was identified in the conditioned medium of fibroblasts as a tryptophan metabolite, 5-methoxytryptophan (5-MTP). It is synthesized via two enzymatic steps: tryptophan hydroxylase (TPH) and hydroxyindole O-methyltransferase (HIOMT). A truncated HIOMT isoform, HIOMT298, catalyzes 5-MTP synthesis. Cancer cells produce scarce 5-MTP due to defective HIOMT298 expression. 5-MTP inhibits cancer cell COX-2 expression and thereby reduces COX-2-mediated cell proliferation and migration. 5-MTP also inhibits MMP-9 expression and thereby reduces cancer cell invasion. 5-MTP exerts its anti-cancer effect by blocking p38 MAPK and p38-mediated NF-κB and p300 HAT activation. The stable transfection of A549 cells with HIOMT298 restores 5-MTP production which renders cancer cells less aggressive. The implantation of HIOMT-transfected A549 into subcutaneous tissues of a murine xenograft tumor model shows that HIOMT-transduced A549 cells form smaller tumors and generate fewer metastatic lung nodules than control A549 cells. HIOMT298 transfection suppresses aromatic amino acid decarboxylase (AADC) expression and serotonin production. Serotonin is a cancer-promoting factor. By restoring 5-MTP and suppressing serotonin production, HIOMT298 overexpression converts cancer cells into less malignant phenotypes. The analysis of HIOMT expression in a human cancer tissue array showed reduced HIOMT levels in a majority of colorectal, pancreatic, and breast cancer. HIOMT298 may be a biomarker of human cancer progression. Furthermore, 5-MTP has the potential to be a lead compound in the development of new therapy for the chemoprevention of certain cancers such as hepatocellular cancer.

## 1. Introduction

Cytoguardin is a cyclooxygenase-2 (COX-2) suppressing factor which was discovered in the conditioned medium (CM) of proliferating fibroblasts (P-Fb) [1,2]. It was noted that COX-2 expression in P-Fb was reduced when compared to that in quiescent fibroblasts (Q-Fb) due to release of a suppressing factor into the CM [2]. The analysis of a purified fraction of the CM by NMR revealed small molecule weight compounds with an indole moiety [2]. As the indole-containing molecules in CM were considered to defend against cellular and tissue damage by COX-2 overexpression, they were named cytoguardins [2]. Cytoguardin activity was not detected in the CM of several cancer cell lines including A549 lung cancer cells, HCT29 colorectal cancer cells, HepB2 hepatocellular cancer cells, and MCF7 breast cancer cells. This finding offers an opportunity to use comparative metabolomics to resolve the chemical identity of cytoguardin. A distinct peak on mass spectrometry is present in the extract of P-Fb CM, but not in that of A549 cancer cells [2]. Investigations with biochemical and molecular genetic approaches identified this peak as 5-methoxytryptophan (5-MTP) [3]. Chemo-synthetic L-5MTP inhibits COX-2 expression in a manner resembling endogenous cytoguardin. Thus, 5-MTP is a key chemical component, if not the only component, of cytoguardin.

5-MTP is produced not only in fibroblasts but also in vascular endothelial cells (ECs), smooth muscle cells (SMCs), and bronchial and renal epithelial cells [4]. Its synthesis is catalyzed by tryptophan hydroxylase (TPH) which converts L-tryptophan to 5-hydroxytryptophan (5-HTP) and hydroxyindole O-methyltransferase (HIOMT) which converts 5-HTP to 5-MTP [3]. 5-MTP is secreted via the Golgi vesicular transport system [4]. 5-MTP acts in a paracrine and autocrine manner to control cancer cell COX-2 expression, cancer cell migration, and cancer growth and metastasis [3]. In addition, it defends against macrophage activation and the release of cytokines [4], and protects the vascular endothelium from damage and leaking [5,6]. Its multitude of actions are mediated by common mechanisms including the inhibition of the p38 MAPK signaling pathway and NF-κB and p300 transcriptional activation [4,7]. Its anti-inflammatory and vasoprotective actions were summarized in a recent review [8]. This review will focus on the anti-tumor actions of 5-MTP. It will summarize the defects of HIOMT expression and deficiency of 5-MTP production in cancer cells, and the metabolic switch and restoration of 5-MTP production and control of cancer cell COX-2 expression and cancer growth and metastasis by HIOMT stable transfection. The mechanisms by which 5-MTP inhibits cancer cell COX-2 expression and reduces cancer growth and metastasis are discussed.

## 2. Deficient Cancer Cell 5-MTP Production Due to Defects of HIOMT Expression

The quantitative analysis of 5-MTP levels by liquid chromatography-mass spectrometry (LC-MS) or enzyme-immunoassay detects very low 5-MTP level in cancer cell CM. As cancer cells express abundant TPH-1 but are unable to convert 5-HTP to 5-MTP, reduced 5-MTP levels are attributed to the defective expression of HIOMT [3]. HIOMT was identified in pineal tissues as the final enzymatic step in melatonin biosynthesis [9]. It catalyzes the conversion of N-acetylserotonin (N-acetyl 5-hydroxytryptamine) to melatonin (N-acetyl 5-methoxytryptamine), and hence it is commonly known as N-acetylserotonin methyltransferase (ASMT). Human ASMT was reported to be encoded by a single gene [10]. However, three mRNA isoforms are detected in pineal cells: isoform 373, which codes for a 373-amino acid (aa) protein, is considered to be the full-length isoform while isoforms 345 and 298 are spliced products in which exon 6 and exon 6 & 7 are spliced, respectively [11]. There is suggestive evidence that isoform 345 is a wild-type ASMT (HIOMT). First, exon 6 in isoform 373 contains repeat LINE-1 sequences and is considered as an insertion from transposons [11]. It is thus possible that isoform 373 may be an insertion mutant. The HIOMT373 isoform is not expressed in other mammalian cells. In fact, bovine and macaque pineal cells express a single mRNA which aligns with the human HIOMT345 isoform. Mouse and rat cells also express a single HIOMT mRNA which aligns with human HIOMT345, but the sequence is more divergent than that of bovine or macaque. Sequence comparison suggests that the 345 isoform is a conserved isoform for melatonin biosynthesis. This is supported by structure-function analysis. Bovine and macaque pineal cells express only ASMT345 which shares with human ASMT345 a high degree of sequence identity. Human 345 isoform was reported to be catalytically active in melatonin synthesis, while isoform 298 and 373 are inactive [12]. ASMT345 is the predominant isoform detected in human pineal and retinal cells. Although ASMT373 and 298 are also detected, albeit at a low level, their functional roles are unclear.

Human fibroblasts and ECs express a single HIOMT isoform which is identical to pineal cell ASMT298 [13]. Cancer cells express a minute quantity of HIOMT298 [13]. The analysis of HIOMT proteins in human cancer tissue arrays reveals a low level of HIOMT in a majority of colorectal (CRC), pancreatic, and breast cancer tissues [13]. It is unclear why cancer cells are defective in HIOMT expression. This could be due to aberrant promoter function or mRNA instability. It is clear, however, that HIOMT298 is active in catalyzing 5-MTP synthesis as stable transfection of A549 cells and with HIOMT298 restores HIOMT expression and 5-MTP production in A549 cancer cells [13]. A deficiency of 5-MTP in cancer cells due to HIOMT expression defects contributes to cancer cell migratory and proliferative activities.

## 3. HIOMT-Transfected Cancer Cells Undergo a Metabolic Switch from Serotonin to 5-MTP Production

Quantitative analysis of 5-HTP metabolites in A549 CM reveals abundant serotonin but scarce 5-MTP and undetectable melatonin [13]. Serotonin was reported to promote growth of hepatocellular, breast, and prostate cancer [14,15]. Serotonin promotes cancer growth by binding to selective receptors, notably 5-HT receptor 2B, via which it activates ERK [16]. Serum serotonin was reported to be a poor prognostic biomarker of breast cancer [17]. Serotonin stimulates prostate cancer cell growth via 5-HT receptor 1A, 1B, 2B, and 4 [18,19,20]. It has been reported that serotonin receptor antagonists inhibit prostate cancer growth [18,19,20]. Serotonin appears to play a dual role in CRC development. It was reported to be crucial for CRC cancer growth by inducing angiogenesis [21]. On the other hand, it protects mouse colonic crypt from DNA damage and CRC tumorigenesis [22]. 

**Figure 1:**
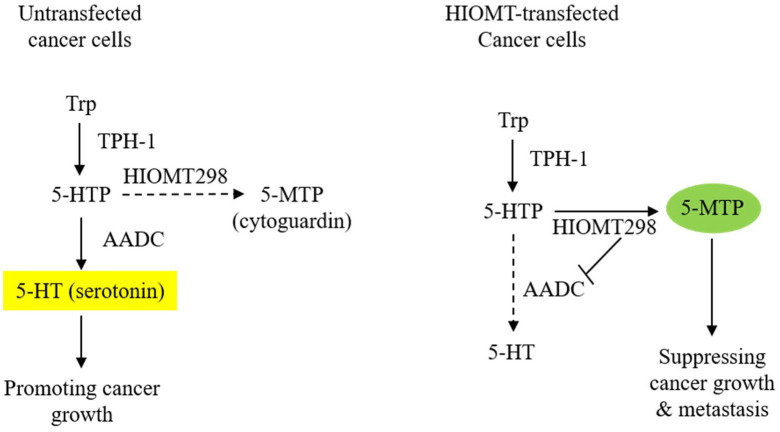
Transfection of A549 cells with HIOMT298 switches 5-hydroxytryptophan (5-HTP) catabolism. The left panel shows AADC/serotonin as the major pathway in untransfected A549 cells and the right panel shows the switch of 5-HTP metabolites from serotonin to 5-MTP synthesis due to the suppression of AADC in HIOMT298-overexpressed A549 cells. Dotted lines denote reduced reaction.

HIOMT298 transfection of A549 cells results in a drastic change in the metabolic profile of the CM. The 5-MTP level becomes highly elevated while serotonin is suppressed and melatonin remains undetectable [13]. Metabolite changes in the CM are correlated with alteration in the enzyme expression: abundant HIOMT298 and diminished expression of aromatic L-amino acid decarboxylase (AADC) (Figure 1). AADC catalyzes the conversion of 5-HTP to 5-hydroxytryptamine (5-HT, serotonin). It is also known as L-DOPA decarboxylase (DDC) as it converts L-DOPA to dopamine [23,24,25]. AADC is expressed in the neurons and cells of peripheral tissues. The neuronal and non-neuronal expression of AADC is driven by distinct promoters due to alternative splicing [26]. Two AADC transcripts are identified which code for AADC 480 and AADC 442 proteins [27]. AADC480 is catalytically active in serotonin synthesis. Cancer cells of neuroendocrine origin such as small cell lung cancer cells (SCLC) express a high level of neuronal type AADC [28]. A549 cells which are non-neuroendocrine cells express a lower level of AADC than SCLC cells; non-neuronal type AADC mRNA is predominant [28]. AADC mRNA levels are reduced by HIOMT298 transfection [13]. It is unclear how HIOMT298 transfection elicits a suppressing effect of AADC expression. It was reported that AADC expression driven by non-neuronal promoters is regulated by hepatocyte nuclear factor 1 (HNF-1) [29]. It remains to be determined whether HIOMT298/5-MTP suppresses AADC expression via this regulatory transcriptional mechanism. The metabolic switch exerts a great influence on cancer cell malignant behavior. It enhances the anti-tumor effect of 5-MTP.

## 4. HIOMT298 Transfection Reduces Cancer Growth and Metastasis via 5-MTP

A549 cells implanted into the subcutaneous tissue of a murine xenograft tumor model grow with time and by seven weeks after implantation they form a sizable tumor with lung metastasis [3,13]. A549 cells with HIOMT298 overexpression via stable transfection exhibit a slower growth rate when implanted in the murine tumor model. The tumor size at seven weeks after HIOMT298-A549 cancer cell implantation is about 50% of that of untransfected A549 cells [13]. By contrast, A549 cells transfected with vectors alone show a faster growth and more lung metastatic nodules than HIOMT-transfected A549 cells [13]. The in vivo data confirm that HIOMT-transfected A549 cells are less malignant.

The intraperitoneal administration of 5-MTP three times a week to the xenograft tumor model implanted with untransfected A549 cells reduces tumor growth rate and tumor volume by about 50% at seven weeks after A549 cell implantation when compared to the vehicle control [3]. 5-MTP administration reduces lung metastasis. The reduction of tumor volume and lung metastatic nodules by 5-MTP is comparable to that of HIOMT298 transfected A549 cells, suggesting that the cancer growth and metastasis of implanted HIOMT298-transfected A549 cells are controlled by cancer cell-produced 5-MTP.

## 5. 5-MTP Inhibits Cancer Growth and Metastasis through the Control of COX-2 Expression

COX-2 is constitutively expressed in many types of human cancers. Constitutive COX-2 expression in cancer cells is driven by complex mechanisms [30]. Several studies have shown that cancer cells exhibit increased β-catenin/T cell factor binding and β-catenin-mediated COX-2 promoter activity [31,32]. The upregulation of pontin52 was implicated as an endogenous driver of β-catenin transactivation [33]. Pontin52 is a nuclear protein which interacts with β-catenin and binds to the TATA box binding protein [34]. Human cancer expresses high levels of pontin52, via which β-catenin mediated COX-2 expression is enhanced. p53 has been implicated in COX-2 expression in cancer cells; p53 upregulates COX-2 expression in cultured colon and breast cancer cells at the transcriptional level [35]. Constitutive COX-2 expression in cancer cells could also be due to increased COX-2 mRNA stability in cancer cells as a result of an increased expression of HuR which stabilizes COX-2 mRNA and increases COX-2 mRNA translation [36].

COX-2 expression in cancer cells is augmented by stimulation with phorbol 12 myristate 13 acetate (PMA), pro-inflammatory cytokines, and growth factors. The COX-2-inducing factors enhance COX-2 transcription via the activation of transactivators such as NF-κB, C/EBPβ, AP-1, and CREB-2, accompanied by the increased binding of transcriptional co-activator p300 and p300 HAT activity [37,38,39]. Cancer cell COX-2 expression is further augmented by factors produced by host stromal cells in the tumor microenvironment [40].

### 5.1. COX-2 Overexpression Drives Cancer Growth and Metastasis

Numerous studies have provided evidence for the important pathogenic role that COX-2 plays in cancer progression. COX-2 overexpression in cancer cells has been shown to confer resistance to apoptosis, promote proliferation, induce angiogenesis, and enhance cellular migration and invasion. Most of the tumor-promoting actions of COX-2 are ascribed to its major metabolite, prostaglandin E_2_ (PGE_2_) [41]. PGE_2_ was reported to promote cancer cell proliferation through the trans-activation of epidermoid growth factor receptor (EGFR) [42]; induce angiogenesis via the HIF-1α/VEGF axis [43]; confer apoptosis resistance via Bcl2, p53 and c-Myc [44,45,46]; and enhance cancer cell migration and invasion via pro-inflammatory mediators [47]. The inhibition of COX-2 activity in cancer cells with selective COX-2 inhibitors results in reduced tumor growth in animal models and human clinical trials [48,49]. The genetic silencing of COX-2 in breast cancer cells was reported to block cancer cell migration and invasion, reducing cancer metastasis in a mouse model [50]. Taken together, these reported data indicate that COX-2 expression is causally correlated with cancer growth and metastasis.

Cancer aggressiveness is augmented by COX-2/PGE_2_-mediated immune suppression. PGE_2_ induces regulatory T cell (Treg) generation through the expression of indole 2,3-dioxygenase (IDO) or tryptophan 2, 3-dioxygenase (TDO) in cancer cells [51,52]. IDO and TDO catalyze kynurenine (Kyn) generation from L-tryptophan. Kyn not only induces Treg but also promotes cancer growth and invasion via the aryl hydrocarbon receptor [40,53].

### 5.2. 5-MTP Inhibits Cancer Cell COX-2 Expression and Cancer Progression and Metastasis

5-MTP inhibits COX-2 expression in A549 cancer cells in a concentration-dependent manner [3]. The effects of 5-MTP on cancer cell COX-2 expression and cancer growth have been evaluated in a murine xenograft tumor model. Intraperitoneal administration of 5-MTP reduces tumor growth and at seven weeks after the subcutaneous implantation of A549 cells, tumor volume was reduced by 50% when compared to the vehicle control [3]. Metastatic lung nodules are reduced by 5-MTP. The analysis of COX-2 in subcutaneous cancer cells reveals the suppression of COX-2 expression in 5-MTP-treated mice. 5-MTP is effective in controlling cancer growth and metastasis through the reduction of COX-2 expression.

### 5.3. 5-MTP Inhibits COX-2 Transcription by Blocking p300 HAT Activation and Transactivator Binding

It is unclear how 5-MTP inhibits cancer cell COX-2 expression. However, it is known that 5-MTP inhibits PMA-induced COX-2 expression in fibroblasts by blocking the binding of NF-κB, C/EBPβ, AP-1, and CREB to the COX-2 promoter [7]. Furthermore, 5-MTP inhibits p300 HAT activity whereby it amplifies its inhibition of COX-2 transcription. It was reported that cancer cells exhibit aberrant autonomous activation of NF-IL6 and CRE [30]. It is possible that 5-MTP inhibits cancer cell COX-2 expression by controlling the binding of C/EBPβ and CREB to the NF-IL6 and CRE cis-acting elements on the promoter of COX-2 and related genes.

## 6. Fibroblasts Inhibit Cancer Cell Migration and Epithelial Mesenchymal Transition (EMT) via 5-MTP

**Figure 2:**
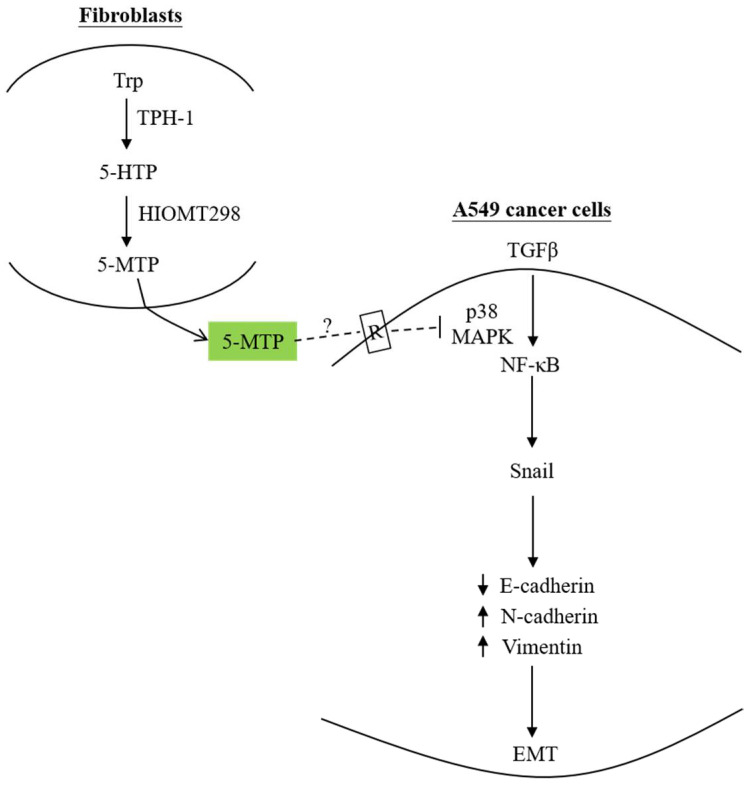
Fibroblast-produced 5-MTP controls A549 EMT. 5-MTP produced by and released from human fibroblasts blocks TGFβ-induced A549 EMT by inhibiting p38 MAPK-mediated NF-κB transactivation, thereby suppressing Snail expression, resulting in reduced E-cadherin and increased *N*-cadherin and vimentin. It was postulated that 5-MTP binds to a cell surface receptor and induces signal transduction which interferes with the p38 MAPK activation pathway. The dotted lines denote a hypothetic view. As the 5-MTP receptor has not been isolated and characterized, further studies are needed to provide evidence to support this hypothesis. Up arrows denote upregulation whereas down arrows, downregulation.

Although it is well recognized that cancer cells are capable of “educating” fibroblasts in the tumor microenvironment and converting them into cancer-promoting cells, i.e., cancer-associated fibroblasts (CAF), there is evidence that naïve fibroblasts possess powerful arsenal to fight cancer [54,55,56,57]. One of these arsenals is 5-MTP. The co-incubation of human fibroblasts with A549 cancer cells in a two-chamber system diminished transforming growth factor-β (TGF-β)-induced A549 cell migration and invasion [58]. Furthermore, co-incubated fibroblasts inhibited TGFβ-induced EMT by suppressing Snail expression and the consequent suppression of *N*-cadherin and vimentin and preservation of E-cadherin. The silencing of TPH-1 in fibroblasts resulted in the reduction of 5-MTP and the abrogation of the anti-migratory and anti-EMT actions of fibroblasts [58]. These findings indicate that 5-MTP is a key factor employed by fibroblasts to control cancer cell migration and EMT. This notion is strengthened by the demonstration that the addition of 5-MTP to A549 cells blocks EMT in a concentration-dependent manner [58]. The effect of 5-MTP on inhibiting EMT is independent of COX-2. However, it is mediated via an NF-κB transcriptional pathway. NF-κB was reported to play an essential role in EMT by upregulating the EMT master regulator Snail [59]. 5-MTP controls TGFβ-induced EMT by inhibiting NF-κB activation, thereby abrogating Snail expression and down-regulating N-cadherin and vimentin expression [58] (Figure 2).

## 7. 5-MTP Controls Cancer Cell EMT and Migration by Interrupting p38 MAPK Pathway

The mechanisms by which 5-MTP inhibits COX-2 overexpression and cancer growth and metastasis are not entirely clear. However, there is strong evidence that 5-MTP exerts its anti-inflammatory and anti-cancer actions by blocking the p38 MAPK pathway. Four p38 MAPK isoforms are identified in mammalian cells, of which p38α is abundantly expressed in most cells while p38β is expressed at low levels. p38γ and p38δ expressions are restricted [60]. Multiple p38 isoforms mediate subunit specific functions. Most reports address the role of p38α isoforms in tumorigenesis and inflammation [61]. p38 MAPK mediates cellular responses to diverse stresses including inflammatory, oxidative, hypoxic, and oncogenic stresses [62,63,64,65]. Furthermore, it mediates stress-induced COX-2 expression in A549 cells [66,67]. Recent reports indicate that as cancer grows, p38 MAPK plays a major role in promoting cancer cell proliferation and migration and increasing cancer metastasis [60]. It was reported that TGF-β-induced A549 EMT signals via p38 MAPK [65,68,69]. 5-MTP inhibits TGFβ-induced p38 MAPK [58]. As p38 MAPK inhibitors block TGFβ-induced A549 EMT, it is likely that 5-MTP inhibits EMT at least in part by blocking p38 MAPK. This is in keeping with reports that 5-MTP targets the p38 MAPK pathway for its vasoprotective and anti-inflammatory actions [8]. 5-MTP protects endothelial VE-cadherin and barrier functions by blocking p38 MAPK activation [6]. It inhibits LPS-induced macrophage activation and cytokine-induced vascular smooth muscle proliferation and migration via suppressing the p38 MAPK pathway [4,70]. p38 MAPK has been considered as a target for cancer treatment and several p38 MAPK inhibitors have been evaluated for their anti-cancer effects in clinical trials. There is only limited success due largely to off-target side effects [60]. 5-MTP has potential utility as a supplement to attenuate cancer growth and metastasis via p38 MAPK.

It is unclear how 5-MTP inhibits stress-induced p38 MAPK activation. One possibility is that 5-MTP acts by binding to its receptors on the cell surface via which signals are transmitted to disrupt the p38 MAPK pathway. It has been suggested that the macrophage membrane may express 5-MTP receptors [4]. However, the receptor has not been isolated and characterized and its signaling pathway has not been unraveled. It remains to be discerned whether 5-MTP acts directly on p38 MAPK or its upstream signaling kinases such as TAK-1 [71] or signaling scaffolds such as lysine-63 (K63)-linked polyubiquitin which plays an essential role in stress-induced signaling and gene expression [72].

## 8. Conclusions

Cytoguardin (5-MTP) is a new tryptophan metabolite with cancer-modulating properties. As opposed to the cancer-promoting effects of serotonin and kynurenine [14,73], 5-MTP inhibits cancer growth and metastasis by suppressing COX-2 and MMP expressions. COX-2 is considered as a key driver of cancer cell migration, invasion, and metastasis. By inhibiting COX-2 expression, 5-MTP reduces cancer growth and metastasis. 5-MTP is also active in blocking cancer cell EMT, a key process promoting cancer metastasis. Thus, 5-MTP is a new class of endogenous metabolite which defends against cancer metastasis.

Fibroblasts in tumor microenvironments are heterogenous. Some are educated by cancer cells to support cancer growth. Cancer-educated fibroblasts (commonly known as cancer-associated fibroblasts, CAF) lose 5-MTP-producing capability. However, certain subtype of fibroblasts in the tumor microenvironment, as well as nascent fibroblasts, produce and secrete 5-MTP to control cancer cell COX-2 expression and cancer growth and metastasis. 

A549 cancer cells express abundant TPH-1 but very low levels of HIOMT298 and hence produce very low level of 5-MTP. They express AADC which directs 5-HTP metabolism toward serotonin synthesis. Serotonin promotes cancer growth. The transfection of A549 cancer cells with HIOMT298 restores 5-MTP synthesis and suppresses serotonin production through the inhibition of AADC expression. This metabolic shift enhances the antitumor effect of 5-MTP. HIOMT-transfected A549 cells exhibit a less malignant phenotype. When they are implanted into the subcutaneous tissues of a murine xenograft model, they grow at a slower rate than control A549 cells and generate few metastatic lung nodules. Thus, it is feasible to engineer cancer cells to restore 5-MTP production and reduce cancer malignancy by transfecting HIOMT298.

In keeping with its anti-inflammatory actions, 5-MTP blocks cancer growth and metastasis by inhibiting p38 MAPK-mediated NF-κB, C/EBPβ, AP-1, and CREB transactivation. It inhibits p300 HAT activity thereby augmenting its inhibition of COX-2 and MMP expression. NF-κB plays an essential role in promoting cancer cell EMT. 5-MTP blocks EMT by inhibiting NF-κB. 5-MTP exerts its diverse biological actions by targeting the p38 MAPK NF-κB pathway.

HIOMT expression is reduced in a majority of human cancers such as colorectal, pancreatic, breast, and hepatocellular cancers (HCC). Preliminary results suggest that cancer tissue HIOMT is a prognostic biomarker of cancer.

The supplementing of 5-MTP has therapeutic potential for the chemoprevention of certain cancer development. For example, HCC development is preceded by liver damage and inflammation [74,75]. The administration of 5-MTP at the pre-cancerous stage of liver disease may prevent progression to cancer by controlling inflammation and stellate cell activation. Further studies are needed to prove this concept.

## Data Availability

Not applicable.

## Abbreviations

5-MTP	5-methoxytryptophan
P-Fb	proliferative fibroblasts
Q-Fb	quiescent fibroblasts
EC	endothelial cells
SMC	smooth muscle cells
TPH	tryptophan hydroxylase
5-HTP	5-hydroxytryptophan
HIOMT	hydroxyindole O-methyltransferase
ASMT	N-acetyl methoxytransferase
COX-2	cyclooxygenase-2
HAT	histone acetyltransferase
CRC	colorectal cancer
HCC	hepatocellular cancer
5-HT	5-hydroxytryptamine
AADC	aromatic L-amino acid decarboxylase
DDC	L-dopa decarboxylase
SCLC	small cell lung cancer
HNF-1	hepatocyte nuclear factor-1
EGF	epidermal growth factor
VEGF	vascular endothelial growth factor
PGE_2_	prostaglandin E_2_
IDO	indolamine 2,3 dioxygenase
TDO	tryptophan 2,3 dioxygenase
EMT	epithelial mesenchymal transition
CAF	cancer-associated fibroblast
MMP	matrix metalloproteinase
